# Cholinergic contributions to speech-in-noise processing in Parkinson’s disease

**DOI:** 10.1093/braincomms/fcag384

**Published:** 2026-10-08

**Authors:** Devin L McCaslin, Stiven Roytman, Timothy NeCamp, Batoul Berri, Taylor Brown, Jaimie Barr, Prabesh Kanel, Giulia Carli, Nicolaas Bohnen

**Affiliations:** Department of Otolaryngology, Neurovestibular Laboratory, University of Michigan, Ann Arbor, MI 48109, USA; Department of Radiology, University of Michigan, Ann Arbor, MI 48109, USA; Functional Neuroimaging, Cognitive, and Mobility Laboratory, Department of Radiology, University of Michigan, Ann Arbor, MI 48105, USA; Data Bloom Consulting, Cincinnati, OH 45174, USA; Department of Otolaryngology, Neurovestibular Laboratory, University of Michigan, Ann Arbor, MI 48109, USA; Department of Radiology, University of Michigan, Ann Arbor, MI 48109, USA; Functional Neuroimaging, Cognitive, and Mobility Laboratory, Department of Radiology, University of Michigan, Ann Arbor, MI 48105, USA; Department of Radiology, University of Michigan, Ann Arbor, MI 48109, USA; Functional Neuroimaging, Cognitive, and Mobility Laboratory, Department of Radiology, University of Michigan, Ann Arbor, MI 48105, USA; Department of Radiology, University of Michigan, Ann Arbor, MI 48109, USA; Functional Neuroimaging, Cognitive, and Mobility Laboratory, Department of Radiology, University of Michigan, Ann Arbor, MI 48105, USA; Morris K. Udall Center of Excellence for Parkinson’s Disease Research, University of Michigan, Ann Arbor, MI 48109, USA; Functional Neuroimaging, Cognitive, and Mobility Laboratory, Department of Radiology, University of Michigan, Ann Arbor, MI 48105, USA; Department of Neurology, University of Michigan, Ann Arbor, MI 48109, USA; Department of Radiology, University of Michigan, Ann Arbor, MI 48109, USA; Functional Neuroimaging, Cognitive, and Mobility Laboratory, Department of Radiology, University of Michigan, Ann Arbor, MI 48105, USA; Morris K. Udall Center of Excellence for Parkinson’s Disease Research, University of Michigan, Ann Arbor, MI 48109, USA; Department of Neurology, University of Michigan, Ann Arbor, MI 48109, USA; Neurology Service & GRECC, VA Ann Arbor Healthcare System, Ann Arbor, MI 48105, USA

**Keywords:** speech understanding in noise, cholinergic system, audiometry, positron emission tomography

## Abstract

Speech perception performance in challenging listening environments declines with age and is particularly impaired in Parkinson’s disease, leading to communication difficulties and cognitive strain. Although peripheral hearing loss contributes to these deficits, dysfunction of the central cholinergic system may play a key role. Parkinson’s disease is characterized by early and widespread cholinergic degeneration. Accordingly, further describing the contribution of cholinergic integrity to functional hearing may offer better insight into the mechanistic link between sensory and cognitive decline and inform targeted therapeutic approaches. Twenty-nine individuals with Parkinson’s disease (20 men, nine women; age range 60–76 years) were enrolled in a cross-sectional study conducted at the University of Michigan Functional Neuroimaging, Cognitive and Mobility Laboratory. Participants completed pure-tone audiometry, tympanometry and Quick Speech-in-Noise testing to assess peripheral and functional hearing. Cholinergic system integrity was quantified using [^18^F]-fluoroethoxybenzovesamicol [^18^F]-FEOBV positron emission tomography imaging, and magnetic resonance imaging was used for anatomical co-registration. Statistical parametric mapping identified regions where cholinergic binding correlated with speech-in-noise performance after adjusting for high-frequency pure-tone average thresholds. Multiple regression and hierarchical model comparisons tested independent and interactive effects of peripheral hearing and cholinergic integrity. High-frequency pure-tone average explained 21.6% of the variance in Quick Speech-in-Noise signal-to-noise ratio loss [β = 0.494 (0.151–0.838), *P* = 0.006]. After adjustment for hearing thresholds, reduced [^18^F]-FEOBV binding in auditory, thalamic, temporoparietal and cerebellar regions was significantly associated with greater signal-to-noise ratio loss (cluster-level family-wise error corrected *P* < 0.01). Cholinergic integrity accounted for an additional 27.5% of the variance beyond peripheral hearing [F = 15.596, *P* < 0.001; β = −0.533 (−0.811 to −0.256), *P* = 0.001]. An interaction between hearing thresholds and cholinergic integrity explained a further 12.7% of the variance (F = 9.671, *P* = 0.005), showing that higher cholinergic integrity mitigated the detrimental effects of hearing loss on speech-in-noise understanding. Observed effects were found to be independent of age and global cognition in the *post hoc* confounder regression analysis. Central cholinergic integrity is a major determinant of speech-in-noise performance in Parkinson’s disease, acting both independently and interactively with peripheral hearing function. These findings indicate that speech-in-noise deficits may reflect early subcortical and cortical cholinergic dysfunction rather than only peripheral auditory decline. Recognition of this relationship underscores the potential for cholinergic-enhancing or neurostimulation-based therapies to improve communication and cognitive outcomes in neurodegenerative disorders.

## Teaser

McCaslin *et al*. report that speech-perception-in-noise in Parkinson’s disease patients depends not merely on peripheral auditory integrity but rather on its interaction with central cholinergic integrity. These findings pave the way to novel treatment approaches that target the cholinergic system to improve functional hearing in patients with neurodegenerative conditions.

## Graphical Abstract

**Graphical Abstract:**
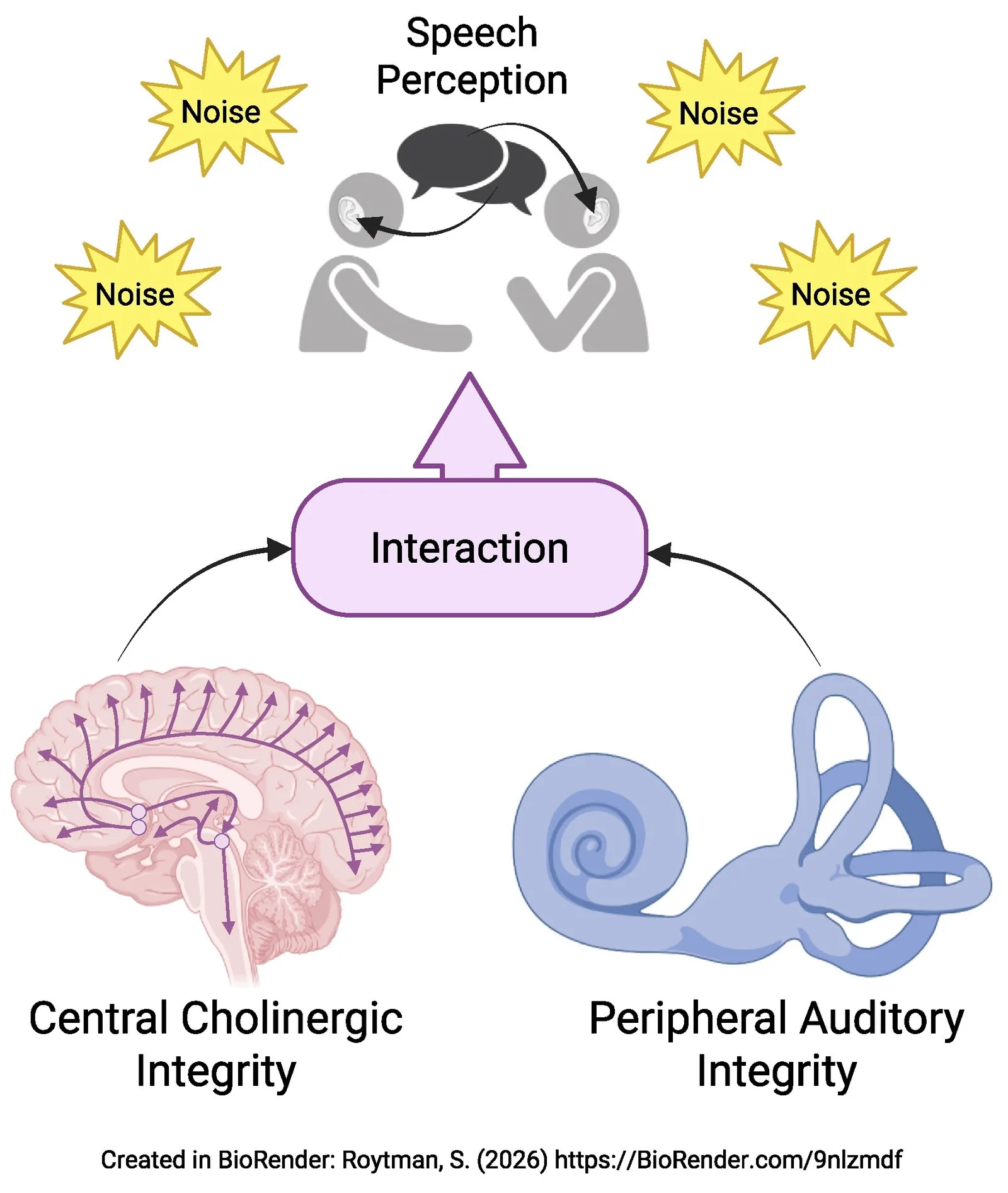

## Introduction

Age-related hearing loss is a widespread public health issue, affecting a significant proportion of older adults.^1^ While the consequences of hearing loss extend beyond communication difficulties, its association with functional decline, social isolation, depression and an increased risk of cognitive decline is well-documented.^2-7^ A growing body of evidence has identified hearing loss as a potential modifiable risk factor for neurodegenerative dementias, emphasizing its critical role in cognitive health.^8^ Current audiologic interventions, such as hearing aids, primarily amplify sound to restore audibility but often inadequately address speech perception in noise (SPIN), and even the most advanced technologies remain limited in this regard. SPIN deficits pose a persistent challenge for ageing individuals, even among a proportion of individuals with normal hearing or mild hearing loss.^9,10^ In individuals with mixed hearing loss (MHL) or sensorineural hearing loss (SNHL), word recognition in quiet (WRQ) scores typically remain excellent until hearing loss reaches a moderately severe degree or worse. By contrast, SPIN performance demonstrates considerable variability and a systematic decline, even with mild hearing impairments. Measures of SPIN have been shown to be sensitive to a wide range of factors related to auditory function decline, both centrally and peripherally, including the presence of vestibular schwannoma,^11^ objective,^10,12^ and perceived hearing impairment.^13^ This divergence between speech understanding in quiet and noise underscores the greater sensitivity of SPIN measures for detecting suprathreshold auditory deficits.^11^

One of the key central neural systems implicated in speech processing and the ability to hear clearly in noisy situations is the cholinergic system (Fig. 1A and B).^14^ The cholinergic system plays a vital role in modulating sensory processing, attention and neural plasticity. The cholinergic basal forebrain projects extensively to the auditory cortex, thalamus and hippocampus and has been shown to enhance the signal-to-noise ratio (SNR) of neural responses, thereby facilitating speech perception under challenging conditions.^15^ In addition, the pedunculopontine nucleus and lateral dorsal tegmental complex provide the major source of cholinergic innervation to the thalamic complex, including the medial geniculate nucleus as well as the inferior colliculus.^15^ It is crucial for top-down as well as bottom-up modulation of auditory processing, as it helps refine sensory input and allocate attentional resources to relevant stimuli.^16^

**Figure 1:**
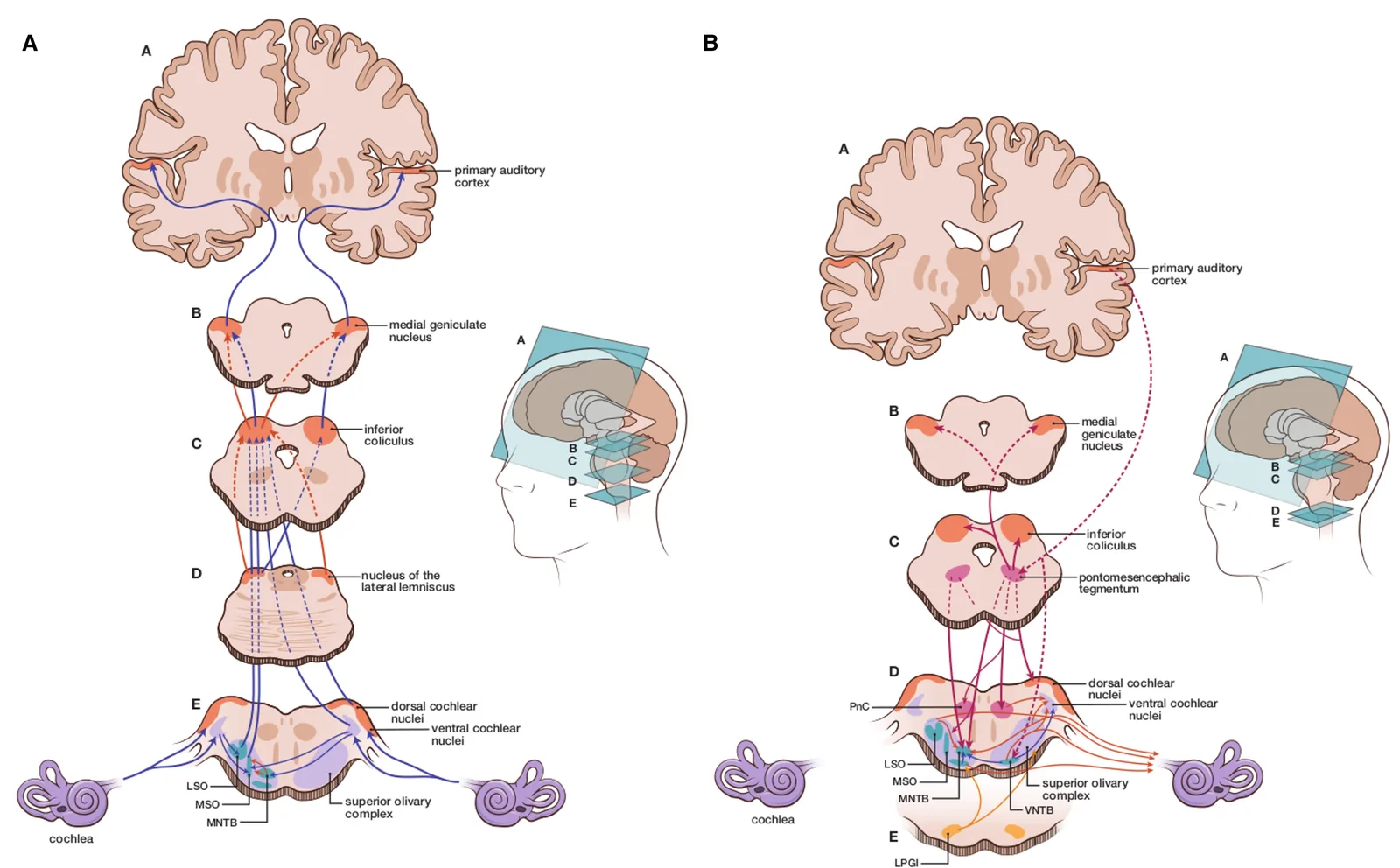
**Ascending auditory pathways and their cholinergic inputs.** Heading A: Illustration of the primary structures in the ascending auditory system. (**A**) Illustration of the primary structures in the ascending auditory system, beginning at the cochlea and extending to the auditory cortex. Excitatory projections are shown with purple arrows, while inhibitory connections are portrayed using orange. Heading B: Illustration of the known cholinergic inputs within the auditory pathway. (**B**) Illustration of the known cholinergic inputs within the auditory pathway. Arrow colours correspond to cholinergic sources, and arrow thickness reflects the relative strength of projections as described by Zhang and Burger 2024. Abbreviations: PnC, pontine reticular nucleus; VNTB, ventral nucleus of the trapezoid body. LPGi, lateral paragigantocellular nucleus; LSO, lateral superior olive; MSO, medial superior olive; MNTB, medial nucleus of the trapezoid body. *Adapted from:* Zhang C, Burger RM. Cholinergic modulation in the vertebrate auditory pathway. *Frontiers in Cellular Neuroscience.* 2024;18:1414484. Licensed under CC BY 4.0.

Compared to normal ageing^17^ and even Alzheimer’s disease, cholinergic degeneration in Parkinson’s disease is more severe and extensive, notably involving the auditory cortices.^18^ Electrophysiological studies further support the role of the cholinergic system in auditory function. For example, Nojszewska *et al*. demonstrated that Parkinson’s disease patients with cognitive impairment exhibited significant abnormalities in auditory evoked potentials, particularly in the P300 response, which is associated with cognitive processing of auditory stimuli.^19^ Such findings suggest that disruptions in cholinergic signalling contribute not only to cognitive impairment but also to central auditory dysfunction in Parkinson’s disease. Moreover, Parkinson’s disease patients often show impaired middle-latency responses, indicative of deficits in brainstem and subcortical auditory processing, further reinforcing the role of the cholinergic system in maintaining auditory function.^20^

An emerging body of evidence indicates a significant association between hearing loss and cognitive function, suggesting that suprathreshold auditory deficits may exacerbate underlying neurological vulnerabilities.^21^ The integrity of the brain’s cholinergic system has been closely associated with both auditory processing and cognitive impairments in Parkinson’s disease.^22-24^ While composite auditory scores were not found to be significantly different relative to control participants, Parkinson’s disease patients demonstrated a moderate strength correlation between auditory integrity and cognition,^25^ suggesting that disease-related central processes may play a critical role in hearing impairment among Parkinson’s disease patients.^26^ Recent research has shown progressive degeneration in cholinergic neurons within the basal forebrain, which significantly disrupts auditory-cognitive network functioning, impairing the ability to effectively segregate speech from background noise and increasing the cognitive load during auditory perception tasks.^27^ The cholinergic system’s broader involvement, particularly its interaction with dopaminergic networks affecting executive and attentional processes, further suggests that disruptions in cholinergic neurotransmission are associated with suprathreshold auditory deficits and also contribute to the wider functional decline observed in Parkinson’s disease.^21^

Given the well-documented role of the cholinergic system in sensory perception and cognitive function, understanding its contributions to SPIN performance is critical for developing targeted interventions such as therapeutics or auditory training. The purpose of this study was to build upon existing research by directly investigating the association between cholinergic system integrity and SPIN performance in persons with Parkinson’s disease. To achieve this, we conducted a cross-sectional analysis using neuroimaging and auditory testing, with the goal of describing how cholinergic dysfunction contributes to SPIN deficits. We hypothesized that cholinergic integrity is a key determinant of SPIN performance, independent of peripheral hearing loss in Parkinson’s disease.

## Materials and methods

### Participants

Twenty-nine participants with Parkinson’s disease (male = 20, age = 68.96 ± 4.5 years) across a spectrum of disease severity (Hoehn and Yahr stages 1–4) were recruited from neurology and audiology clinics at the University of Michigan. To be included, participants had to be at least 50 years old, have no diagnosis of dementia, and be able to provide informed consent. Individuals with Parkinson’s disease had to meet the UK Parkinson's Disease Society Brain Bank Research Center diagnostic criteria. Exclusion criteria included severe cognitive impairment [Montreal Cognitive Assessment (MoCA) score <18], significant medical illness that would prevent study participation (e.g. advanced cardiovascular disease, terminal cancer), untreated psychiatric conditions or contraindications for MRI or PET imaging (e.g. metal implants, claustrophobia). Participants unable or unwilling to complete study assessments were also excluded.

### Measures and procedures

Participants completed detailed clinical evaluations, including the Unified Parkinson’s Disease Rating Scale (UPDRS) to assess motor symptoms in individuals with Parkinson’s disease, along with auditory and neuroimaging assessments completed across two study visits. The first visit consisted of comprehensive audiometric clinical testing, while the second visit included PET and MRI neuroimaging to evaluate the integrity of the central cholinergic system. Dopaminergic medication status was recorded, with levodopa equivalent dose (LED) calculated for medicated Parkinson’s disease participants. All participants with Parkinson’s disease were tested in the OFF medication state following overnight withdrawal of dopaminergic medications (∼12 h). This approach was used to minimize the acute effects of dopaminergic therapy on behavioural performance and to better characterize disease-related deficits.

### Audiometric and SPIN testing

Peripheral hearing function was assessed using pure-tone audiometry at 250 Hz–8 kHz. The high-frequency pure-tone average (HFPTA) was calculated based on thresholds at 1 kHz, 2 kHz and 4 kHz. Middle ear function was assessed via tympanometry to ensure no patients with conductive loss were included in the cohort. SPIN was measured using the Quick Speech-in-Noise (QuickSIN)^28^ test, which evaluates sentence recognition in multi-talker babble across varying SNRs. In accordance with the QuickSIN administration guidelines, each participant completed two practice lists (Lists 21 and 23) to become familiar with both the test environment and task. The order of these practice lists was alternated across listeners. Following practice, QuickSIN lists were presented in randomized order. The total duration of testing for each listener was less than 30 min.

All stimuli were delivered via an Interacoustics Equinox audiometer using ER-3A insert earphones. QuickSIN testing was completed monaurally in each ear using two lists per condition, and the final SNR loss was calculated as the average of the two lists. Each test list contained six sentences embedded in continuous four-talker babble. The background babble remained constant across each list, while the SNR decreased in 5 dB steps. The sentences, spoken by a female talker, were each ∼2.5–3 s long, with 5–6 s intervals between sentences. Each list had a total duration of ∼55 s. Sentence stimuli were presented at a fixed level of 70 dB HL, and the background multi-talker babble was adjusted in 5 dB SNR decrements. Presentation levels were increased when necessary to ensure audibility while remaining below the participant’s reported uncomfortable loudness level. Testing took place in a single-walled sound-attenuating booth, and participants’ verbal responses were recorded directly into a spreadsheet for scoring. Prior to testing, participants received the standard QuickSIN instructions. The primary measure was QuickSIN SNR loss, with higher scores indicating worse performance.

### Neuroimaging

Magnetic resonance imaging (MRI): Structural MRI was performed using a 3T Philips Achieva system to obtain high-resolution anatomical images for brain segmentation and co-registration with PET data. Structural MRI images were segmented using the FreeSurfer software suite. To assess cholinergic system integrity, [^18^F]-FEOBV PET imaging was performed using either a Siemens Biograph Vision 600 scanner or a Biograph 6 TruePoint PET/CT scanner.^29^ Imaging occurred in 3D mode, with a dynamic scan initiated 3 h post-intravenous injection of 8 mCi [^18^F]-FEOBV. Preprocessing of MRI and PET data was performed using previously published procedures,^29^ with specific details provided in the Supplementary materials.

### Statistical analysis

Descriptive statistics were calculated for all measures, including means, standard deviations and ranges. The primary hypothesis that cholinergic system integrity predicts variance in SPIN independent of peripheral hearing loss was tested using a statistical parametric mapping (SPM) approach. First, a regression model was fitted predicting SPIN SNR loss from HFPTA to obtain residual variance, representing the variance in SPIN performance unrelated to peripheral hearing function. Next, a voxelwise correlation was performed correlating [^18^F]-FEOBV with the SPIN residuals. A voxel-level uncorrected significance threshold of *P* < 0.05 was used, followed by a cluster-level familywise error rate corrected threshold of *P* < 0.001.

The resulting statistically significant findings were used to define a mask in study-specific template space, representing voxels where [^18^F]-FEOBV associated significance in SPIN variance unrelated to peripheral hearing loss. The mean DVR from standard space parametric images was extracted using this masked and entered a multiple regression model predicting SPIN from HFPTA and [^18^F]-FEOBV. Hypothesis testing was performed via an *F*-test linear model comparison approach, comparing the model with both HFPTA and [^18^F]-FEOBV as regressors against a model with only HFPTA as a regressor, with an *α* significance threshold set at 0.05. An additional model comparison was performed, comparing a model with an interaction effect between [^18^F]-FEOBV and HFPTA against a model with [^18^F]-FEOBV and HFPTA as independent regressors, to examine whether cholinergic system integrity modulates the impact of peripheral hearing loss on functional hearing ability. Residuals for the final model of best fit were examined and tested for normality using the Shapiro–Wilk test and heteroskedasticity using the Breusch-Pagan test. In case of evidence for deviation from regression modelling assumptions, the model was refitted as a robust linear model, which is less sensitive to the violation of assumptions, to examine the impact on estimated regression coefficients.

A *post hoc* confounder analysis was performed in the sample of Parkinson’s disease patients, adding age and Montreal Cognitive Assessment (MoCA) scores (indicative of general cognitive status) as covariates into the interaction model to ensure that the observed effects were independent of these covariates. To examine whether the cholinergic system topography associated with SPIN performance in Parkinson’s disease patients was disease-specific, the mean value of all statistically significant voxels was extracted from a sample of 13 normal control (NC) patients using the same procedures as for the Parkinson’s disease sample. An ANCOVA analysis was performed, comparing central cholinergic integrity in this pattern of voxels between Parkinson’s disease and NC patients after correction for age. Lastly, the interaction model, as specified in our primary analysis of the Parkinson’s disease patients, was re-estimated on the more limited NC sample, to examine whether any of the effects observed in Parkinson’s disease patients were recapitulated in NC participants.

## Results

Among the 29 Parkinson’s disease participants, nine were females, mean age was 68.97 ± 4.5 years, mean HFPTA decibel threshold was 27.33 ± 11.5 and mean QuickSIN SNR loss was 2.9 ± 2.92. Among the 13 control participants included for the *post hoc* analysis, six were females, mean age was 72.8 ± 8.28 years, mean HFPTA decibel threshold was 34.23 ± 21.69 and mean QuickSIN SNR loss was 3.42 ± 4.44. Group-level HFPTA means (with standard error bars) are visualized by frequency in Fig. 2.

**Figure 2:**
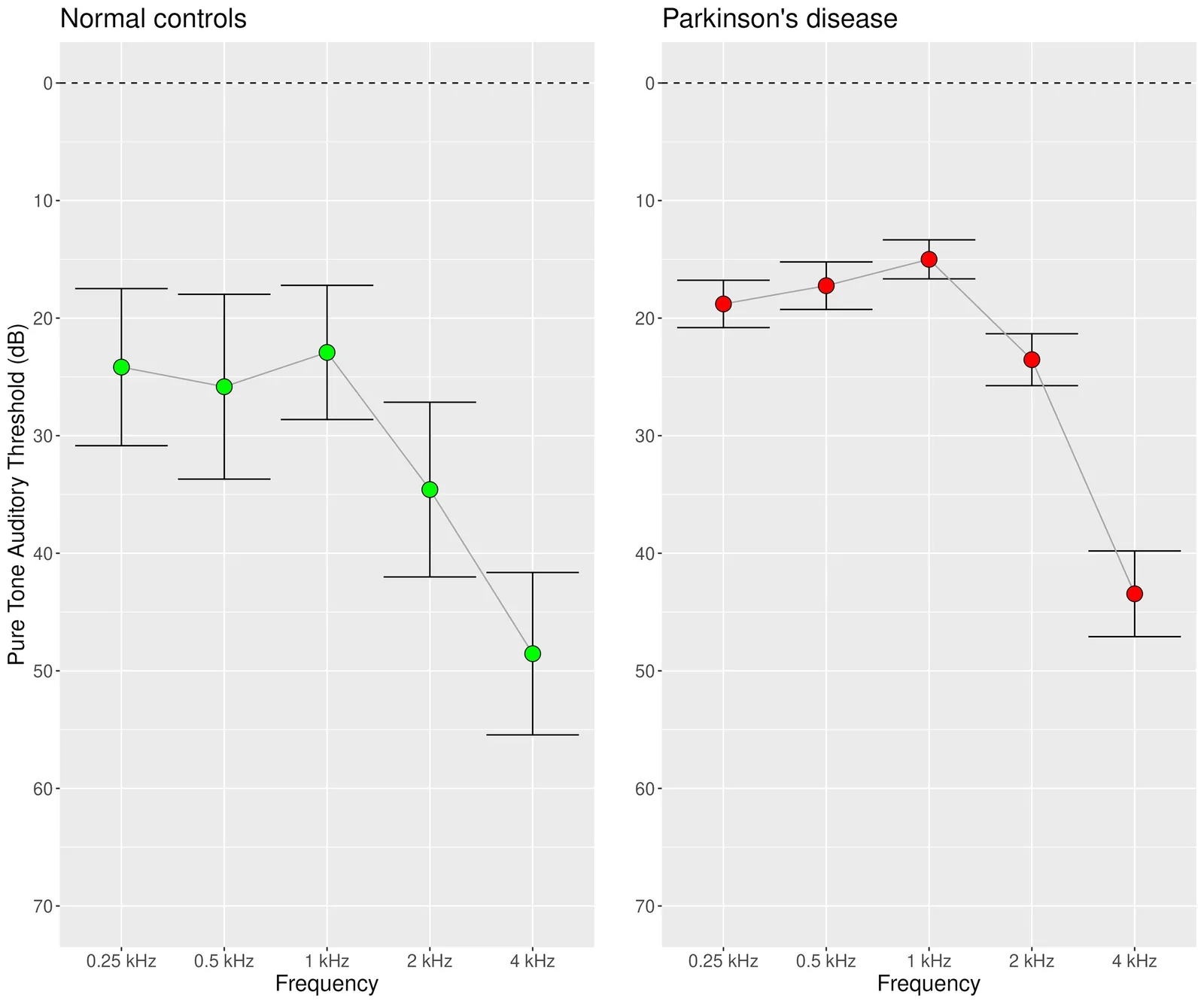
**Group level plot of frequency-specific auditory thresholds.** Group-level audiometric thresholds for the Parkinson’s disease and normal control cohorts. Pure-tone audiometry thresholds are plotted across test frequencies (0.25–8 kHz). Error bars represent standard error of the mean (SEM). Higher thresholds indicate poorer hearing sensitivity.

The regression model predicting QuickSIN SNR loss from HFPTA demonstrated a statistically significant positive association between the two variables [*β* = 0.494 (0.151, 0.838), *P* = 0.006], with poorer peripheral auditory integrity accounting for 21.6% of the variance in speech-in-noise performance. The univariate relationship is visualized in Fig. 3.

**Figure 3:**
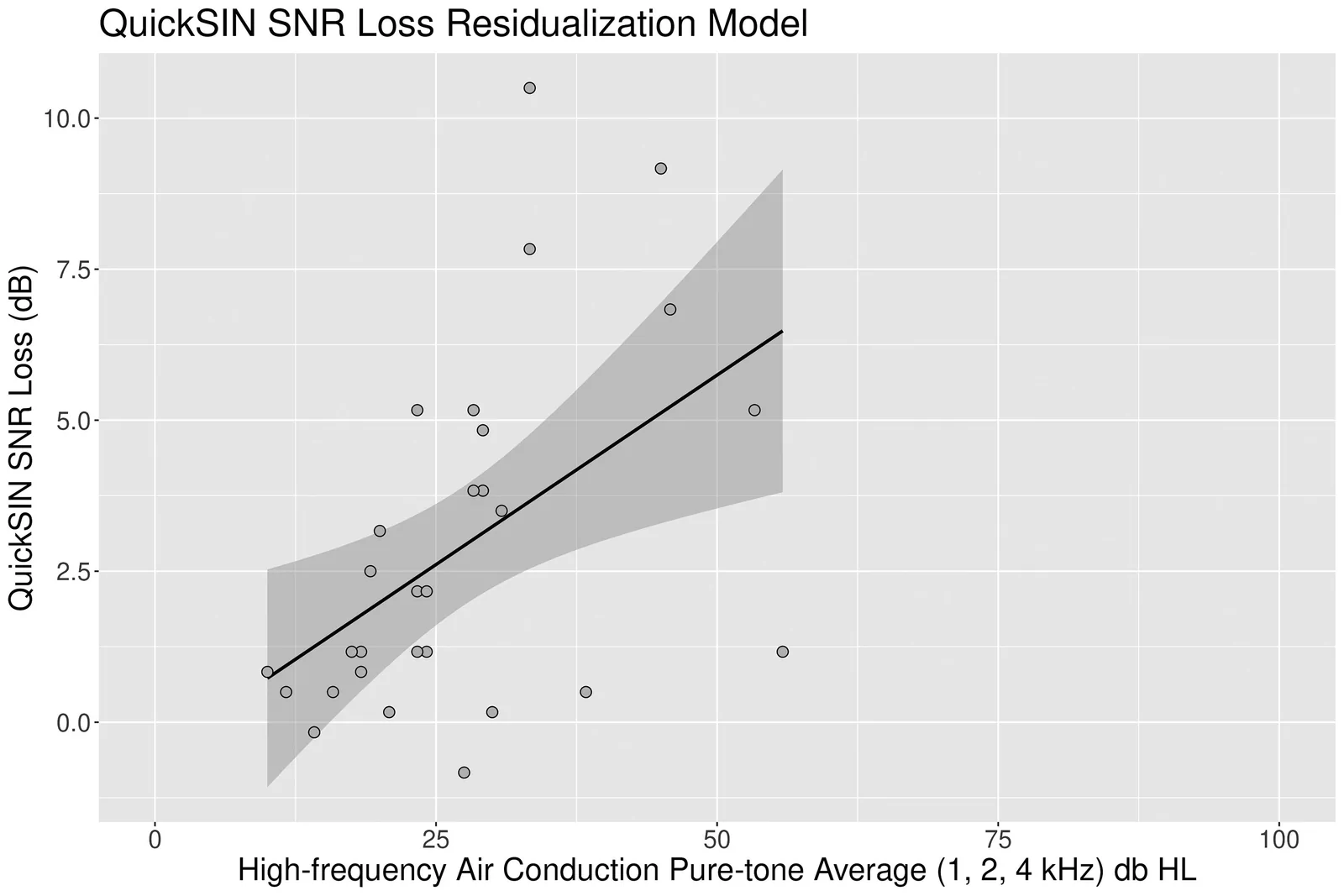
**Relationship between peripheral hearing and speech-in-noise performance.** Scatter plot shows QuickSIN signal-to-noise ratio (SNR) loss versus high-frequency pure-tone average (HFPTA; mean of thresholds at 1, 2 and 4 kHz); higher SNR loss indicates worse performance. Points represent individual participants, and the line depicts the simple linear fit with 95% confidence band. Poorer peripheral hearing (higher HFPTA) was significantly associated with greater SNR loss [*N* = 29, *β* = 0.494 (0.151, 0.838), *P* = 0.006; R^2^ = 0.216]. Statistical significance of the regression model coefficients was assessed using *t*-test.

HFPTA-adjusted residuals were entered into a voxelwise SPM analysis predicting whole-brain [^18^F]-FEOBV uptake, a significant negative correlation emerged between SPIN deficits and cholinergic integrity (see Fig. 4), independent of hearing thresholds, across a range of regions primarily involving occipito-temporo-parietal cortices, left putamen, bilateral cerebellar cortices, left more than right thalamus and left amygdala.

**Figure 4:**
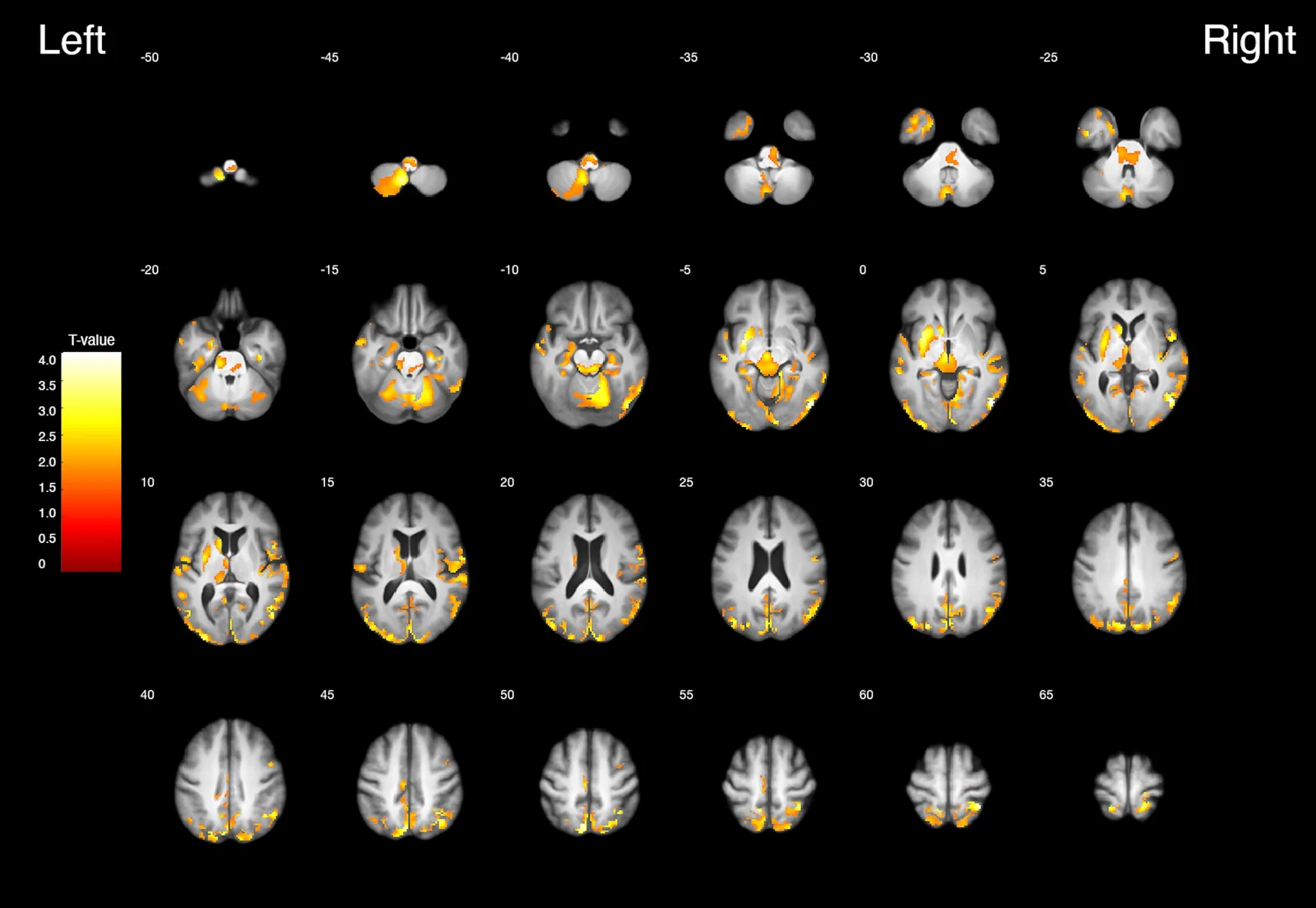
**Whole-brain voxelwise associations between cholinergic binding and speech-in-noise performance (residualized for HFPTA).** Axial slices (MNI z-coordinates, mm) show clusters (yellow) where QuickSIN signal-to-noise ratio (SNR) loss correlates with [^18^F]-FEOBV PET uptake after cluster-level familywise correction for multiple comparisons (*N* = 29, cluster-level FWE *P* < 0.001). Higher SNR loss (worse performance) was linked to lower binding in bilateral temporal/auditory cortex with extensions into thalamic and basal forebrain regions. Overlays are displayed on a study-specific standard space template; the statistical overlay identifies regions showing significant a ssociations, with increasing intensity indicating stronger statistical effects, as indicated by the T-value scale. Abbreviations: [^18^F]-FEOBV, [^18^F]-fluoroethoxybenzovesamicol; QuickSIN, Quick Speech-in-Noise test; MNI, Montreal Neurological Institute; FWE, familywise error; PET, positron emission tomography.

Hierarchical model comparison between the model containing only peripheral auditory integrity and the model containing both peripheral auditory integrity and mean DVR of the statistically significant cluster from the voxelwise analysis as independent predictors of SPIN performance revealed that central cholinergic system integrity explained an additional 27.5% of the variance in SPIN performance independently of peripheral auditory integrity (*R*^2^ = 0.491, *F* = 15.596, *P* < 0.001), with higher cholinergic integrity associating with lower (better) QuickSIN SNR (*β* = −0.533 [−0.811, −0.256], *P* = 0.001). Model comparison between the independent effects model and the interaction model further revealed that the interaction between peripheral auditory integrity and central cholinergic integrity explains an additional 12.7% of the variance in QuickSIN SNR (*R*^2^ = 0.619, *F* = 9.760, *P* = 0.00463). Coefficients of the resulting model suggest that higher pure tone hearing thresholds are independently and positively associated with QuickSIN SNR loss and thus poorer SPIN performance [*β* = 0.722 (0.450, 0.994), *P* < 0.001]. Higher cholinergic system integrity as measured by [^18^F]-FEOBV PET is independently and negatively associated with QuickSIN SNR loss and thus better SPIN performance [*β* = −0.444 (−0.692, −0.196), *P* = 0.001]. Lastly, the interaction effect between pure tone hearing thresholds and central cholinergic system integrity was statistically significant and negative [*β* = −0.456 (−0.758, −0.154), *P* = 0.005], suggesting that higher cholinergic system integrity not only independently associated with better SPIN performance but furthermore ameliorates the adverse impact of higher pure tone hearing thresholds on SPIN performance. The interaction model met both the normality and homoscedasticity assumptions of the residuals.

The interaction model is visualized as a scatterplot of SNR loss over [^18^F]-FEOBV mean cluster DVR with line of best fit split by HFPTA tertile (see Fig. 5), which clearly demonstrates that in the lower and middle tertiles of HFPTA thresholds (better peripheral auditory integrity), the relationship between cholinergic integrity and SPIN performance is modest. The slope of the line of best fit, however, becomes more strongly negative in the upper HFPTA threshold tertile, suggesting that central cholinergic integrity plays a progressively more prominent role in SPIN performance as peripheral auditory integrity deteriorates. All of these findings are in agreement with the obtained model estimates.

**Figure 5:**
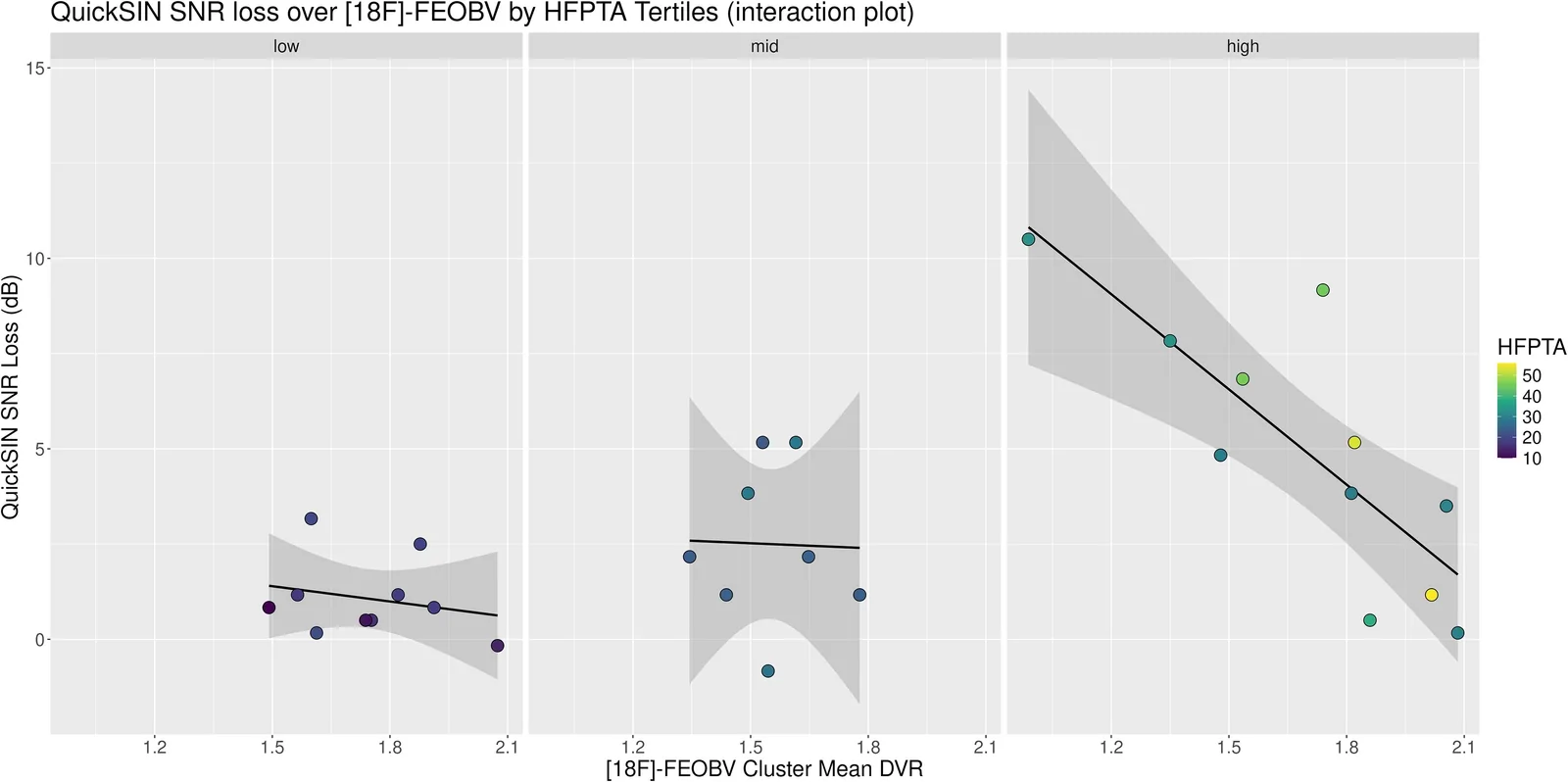
**QuickSIN signal to noise ratio (SNR) loss over cholinergic binding across hearing strata.** Each panel shows participants split by tertiles of high frequency pure tone average (HFPTA: low, mid, high). The *x* axis is the [^18^F]-FEOBV PET cluster mean distribution volume ratio (DVR) and the *y*-axis is QuickSIN SNR loss (higher values indicate worse performance). Points are individual participants (fill colour indicates continuous pure tone hearing thresholds; with brighter colour indicating poorer peripheral auditory integrity). Lines depict simple linear fits with 95% confidence bands. In the high HFPTA group, lower FEOBV binding is associated with greater SNR loss, whereas relationships are weak in the low and mid groups [*N* = 29, *β* = −0.456 (−0.758, −0.154), *P* = 0.005]. Statistical significance of the regression model coefficients was assessed using *t*-test. Abbreviations: [^18^F]-FEOBV, [^18^F]-fluoroethoxybenzovesamicol; DVR, distribution volume ratio.

The confounder analyses revealed that neither age [*β* = 0.066 (−0.204, 0.335), *P* = 0.619] nor MoCA scores [*β* = 0.019 (−0.262, 0.301), *P* = 0.889] were significant independent predictors of SPIN performance in Parkinson’s disease patients. The coefficient estimates for effects that were originally present in the interaction model remained unchanged, suggesting that the effects observed in the primary analysis were not confounded with age and non-specific cognitive changes in the Parkinson’s disease sample.

The ANCOVA model of SPIN-related central cholinergic integrity revealed a weaker trend for a negative association with age [*β* = −0.272 (−0.587, 0.042), *P* = 0.088] and a stronger negative and statistically significant effect of Parkinson’s disease status [*β* = −0.743 (−1.423, −0.062), *P* = 0.033], suggesting that the pattern of cholinergic uptake associated with SPIN in Parkinson’s disease reflects primarily disease-specific rather than age-related changes. Lastly, the original interaction model re-estimated on a limited sample of 13 NC participants revealed a nearly statistically significant marginal effect of HFPTA [*β* = 0.755 (−0.032, 1.540), *P* = 0.058], but no statistically significant marginal effect of central cholinergic integrity [*β* = 0.024 (−0.561, 0.609), *P* = 0.929] nor HFPTA by central cholinergic integrity interaction effect [*β* = −0.123 (−0.687, 0.441), *P* = 0.663]. Taken together, these findings suggest that the interplay between central cholinergic integrity, peripheral auditory integrity and SPIN performance may only be observable under conditions of more severe cholinergic loss in relevant regions, as is the case in Parkinson’s disease.

## Discussion

### Summary of findings and implications

Collectively, these results support the hypothesis that central cholinergic function plays a crucial role in SPIN performance, independent of peripheral hearing loss. The findings further suggest that in healthy ageing, the cholinergic system may serve a compensatory role for age-related hearing decline, whereas in Parkinson’s disease this mechanism is significantly impaired in Parkinson’s disease. When cholinergic integrity is compromised, the challenges individuals with hearing loss face in adverse listening conditions are markedly exacerbated. Thus, the present study provides compelling evidence that central cholinergic system integrity not only exerts an independent influence on SPIN performance but also modulates the contributions of peripheral hearing function. These findings align with prior research demonstrating that central auditory mechanisms contribute significantly to speech processing beyond the effects of peripheral hearing loss.^25^

### Cholinergic system compensation and SPIN performance

The observed interaction between cholinergic integrity and HFPTA in predicting SPIN performance provides novel insights into the compensatory role of the cholinergic system. It is noteworthy that HFPTA accounted for ∼22% of the variance in QuickSIN performance in the present cohort. Prior studies in larger and more heterogeneous clinical populations have reported that hearing thresholds may account for 40–50% of the variance in speech-in-noise performance.^10^ The smaller proportion of variance explained in the current study may reflect the relatively modest sample size and restricted range of hearing thresholds among participants. Alternatively, it may suggest that speech-in-noise performance in Parkinson’s disease is influenced to a greater extent by central factors, including cholinergic dysfunction, beyond what would be expected based on peripheral hearing loss alone. This conclusion is further supported by our failure to observe statistically significant effects of cholinergic integrity and its interaction with peripheral auditory integrity in a more limited sample of controls, amongst whom the variance in cholinergic integrity was relatively limited compared to the Parkinson’s disease patients.

Our findings support the view that central mechanisms can actively offset the loss of afferent auditory input, a phenomenon previously reported in ageing and cognitive decline.^30^ Our findings extend this framework by showing that populations at risk of early cholinergic degeneration, such as Parkinson’s disease patients, face an increased risk of functional hearing decline.^31^ These results are in agreement with prior studies demonstrating that Parkinson’s disease-related cholinergic deficits manifest in various sensory and cognitive impairments, including balance dysfunction and cognitive decline.^32^

Although the current findings highlight a significant association between cholinergic integrity and SPIN independently of global cognition as measured by MoCA scores, integrity of more specific cognitive domains such as attention could potentially be a factor in mediating part of this relationship. The cholinergic system is critically involved in attentional modulation of sensory processing. Previous reports have shown that acetylcholine enhances cortical SNR ratio through selective amplification of behaviourally relevant auditory inputs.^33^ Accordingly, in Parkinson’s disease, compromised cholinergic function may lead to inefficient attentional filtering, increased listening effort and increased cognitive load. To understand the associations between these factors, future investigations should be designed that incorporate measures of auditory attention or cognitive load during SPIN testing. This would be a good first step in the effort to disentangle whether attentional control acts as a mediator of this relationship.

### Hearing loss in Parkinson’s disease

The relationship between hearing loss and Parkinson’s disease further contextualizes the present results. Recent population-based work in *JAMA Neurology* demonstrated that audiometrically confirmed hearing loss is an independent risk factor for incident Parkinson’s disease, with early hearing aid use attenuating this risk.^34^ In a similar study, an East London primary care cohort identified hearing loss and epilepsy as novel diagnostic features up to a decade before Parkinson’s disease diagnosis.^35^ Taken together, these reports suggest that auditory dysfunction may not merely be a prodromal symptom but rather a surrogate marker of underlying cholinergic degeneration. The current investigations’ data support this interpretation that reduced cortical cholinergic integrity corresponded to degraded SPIN performance independent of peripheral hearing thresholds. This supports the idea that hearing loss and impaired auditory perception in challenging situations may be phenotypic expressions of central neurochemical deficits.

These reports in conjunction with the current findings from this investigation point to a model in which central cholinergic decline contributes both to perceptual (hearing and SPIN) and cognitive symptoms of Parkinson’s disease. This framework challenges the traditional view of hearing loss solely as a peripheral sensory disorder and places it in the broader landscape of Parkinson’s disease-related neurodegeneration. Understanding the relationships between auditory processing, attention and cholinergic function challenges conventional thinking as it relates to hearing loss and offers an opportunity for novel diagnostic assessments and therapeutic targets where interventions are designed to restore cholinergic signalling and could potentially improve not only cognition but also functional hearing and communication in everyday environments.

Although HFPTA was used as a clinical estimate of peripheral hearing function, pure-tone thresholds may reflect influences beyond cochlear integrity alone and can be affected by higher-order processes including attention, task demands and cognitive function. Recent investigations have utilized objective peripheral auditory assessments, including otoacoustic emissions, alongside behavioural hearing assessments to better isolate cochlear contributions and characterize auditory dysfunction in Parkinson’s disease and related neurodegenerative conditions.^36,37^ Objective measures of cochlear function may help differentiate peripheral auditory impairment from central auditory and cognitive contributions to speech-in-noise performance. Investigations that incorporate otoacoustic emissions and neuroimaging techniques may be capable of providing a better understanding of the contributions of peripheral and central auditory dysfunction in Parkinson’s disease. Another limitation of the current study is that subcortical auditory processing was not directly assessed. As a result, we cannot exclude the possibility that brainstem-level auditory encoding deficits contributed to the observed associations between cholinergic integrity and speech-in-noise performance. Investigations going forward that incorporate auditory evoked potentials in addition to PET imaging may help inform researchers regarding the contributions of peripheral, subcortical and central cholinergic mechanisms.

The neurochemical mechanisms underlying auditory dysfunction in Parkinson’s disease are likely more complex than isolated central cholinergic degeneration. Cholinergic pathways may influence peripheral hearing through medial olivocochlear (MOC) modulation of cochlear amplifier gain, while dopaminergic dysfunction involving lateral olivocochlear (LOC) pathways may alter auditory nerve activity and increase susceptibility to synaptic dysfunction.^34,38^ Recent work suggests that multiple neurotransmitter systems may contribute simultaneously to auditory abnormalities in Parkinson’s disease and raises the possibility that neurological and auditory dysfunction may influence one another in a bidirectional manner. Although the current study specifically evaluated central cholinergic integrity using [^18^F]-FEOBV PET imaging, future investigations integrating peripheral auditory physiology with additional neurochemical biomarkers included may provide a more complete understanding of these interactions.

In addition to attentional influences, there are other factors that may play a role in the observed association between SPIN and the cholinergic system. Disease duration, stage and medication state are all important covariates that may modify cholinergic contributions to auditory processing. There is no question that cholinergic degeneration progresses nonlinearly across Parkinson’s disease stages, and mapping [^18^F]-FEOBV binding along the disease continuum may provide insight into whether SPIN impairment reflects early cortical cholinergic decline or later multisystem involvement. Tracking the longitudinal characterization of cholinergic system decline could provide a temporal ‘time course’ that connects central auditory integrity with disease progression and potentially identify a prodromal window for intervention.

### Clinical implications and future directions

These findings have important clinical implications for improving hearing healthcare and cognitive screening in older adults and Parkinson’s disease populations. Importantly, the mean QuickSIN performance observed in the present cohort was comparable to values reported by Fitzgerald et al. (2023)^10^ in a large clinical sample of over 5800 patients with similar degrees of hearing loss. This finding suggests that the speech-in-noise performance of our participants was broadly representative of what would be expected in clinical populations with comparable peripheral hearing function, supporting the generalizability of the present findings. Given the role of the cholinergic system in mitigating SPIN deficits, targeted interventions such as pharmacological treatments with cholinesterase inhibitors, muscarinic allosteric modulators or nicotinic receptor agonists, as well as neurostimulation techniques, may offer opportunities to enhance both auditory and cognitive outcomes. Understanding the neurobiological mechanisms underlying SPIN performance may also inform the development of rehabilitative strategies aimed at strengthening central auditory processing. Future studies should investigate whether cholinergic-targeted therapies improve SPIN outcomes and assess the potential of SPIN deficits as an early biomarker of cholinergic dysfunction in ageing and neurodegenerative diseases. Recent evidence suggests that the relationship between SPIN deficits and cognitive function may be mediated by the integrity of the cholinergic integrity, raising the possibility that SPIN testing could serve as a non-invasive and functionally relevant tool for early identification of individuals at risk for cognitive decline. Although cognitive outcomes were not analysed in the present study, future work should explore whether SPIN testing may contribute to earlier diagnosis and intervention in ageing and Parkinson’s disease populations. Lastly, future studies would benefit from the inclusion of subjective measures of listening difficulties,^39^ to draw clearer connections between SPIN deficits and their impact on daily living.

## Conclusion

Overall, the results of this study add to a growing body of evidence highlighting the central role of the cholinergic system in auditory function, particularly under challenging listening conditions. The findings underscore the importance of central auditory processing in SPIN performance and reveal the vulnerability of this system in Parkinson’s disease. By identifying the cholinergic system as a key mediator between SPIN deficits and auditory performance in noise, this study paves the way for new approaches to early detection and sets forth novel ways of approaching treatment for hearing loss. Future research should further examine the mechanistic pathways underlying cholinergic compensation and evaluate the efficacy of targeted interventions aimed at improving hearing in noise in ageing populations.

## Supplementary Material

fcag384_Supplementary_Data

## Data Availability

The dataset analysed in the present article will be made available upon reasonable request to the senior author (Dr Nicolaas Bohnen). The R code used for performing statistical analysis has been made publicly accessible on a GitHub repository (https://github.com/neuro-stivenr/braincomm-spin-analysis).

## References

[1] Reed NS, Garcia-Morales EE, Myers C, et al Prevalence of hearing loss and hearing aid use among US medicare beneficiaries aged 71 years and older. JAMA Netw Open. 2023;6(7):e2326320. 10.1001/jamanetworkopen.2023.26320 PMC10383002 37505496

[2] López-Torres Hidalgo J, Boix Gras C, Téllez Lapeira J, et al Functional status of elderly people with hearing loss. Arch Gerontol Geriatr. 2009;49(1):88–92. 10.1016/j.archger.2008.05.006 18603314

[3] Arjmandi MK, Neils-Strunjas J, Nemati S, et al Age-related hearing loss, cognitive decline, and social interaction: Testing a framework. J Speech Lang Hear Res. 2024;67(8):2743–2760. 10.1044/2024_JSLHR-23-00810 38995870

[4] Li F, Jin M, Ma T, Cui C. Association between age-related hearing loss and depression: A systematic review and meta-analysis. PLoS One. 2025;20(1):e0298495. 10.1371/journal.pone.0298495 PMC11698527 39752630

[5] Martinez-Amezcua P, Powell D, Kuo PL, et al Association of age-related hearing impairment with physical functioning among community-dwelling older adults in the US. JAMA Netw Open. 2021;4(6):e2113742. 10.1001/jamanetworkopen.2021.13742 PMC8233700 34170305

[6] Myrstad C, Engdahl BL, Costafreda SG, et al Hearing impairment and risk of dementia in the HUNT study (HUNT4 70+): A Norwegian cohort study. EClinicalMedicine. 2023;66:102319. 10.1016/j.eclinm.2023.102319 PMC10772264 38192588

[7] Conceição Santos de Oliveira D, Gomes-Filho IS, Araújo EM, et al Association between hearing loss and cognitive decline in the elderly: A systematic review with meta-analysis study. PLoS One. 2023;18(11):e0288099. 10.1371/journal.pone.0288099 PMC10635537 37943811

[8] Powell DS, Oh ES, Reed NS, Lin FR, Deal JA. Hearing loss and cognition: What we know and where we need to go. Front Aging Neurosci. 2022;13:769405. 10.3389/fnagi.2021.769405 PMC8920093 35295208

[9] Ross B, Dobri S, Schumann A. Psychometric function for speech-in-noise tests accounts for word-recognition deficits in older listeners. J Acoust Soc Am. 2021;149(4):2337. 10.1121/10.0003956 33940923

[10] Fitzgerald MB, Gianakas SP, Qian ZJ, Losorelli S, Swanson AC. Preliminary guidelines for replacing word-recognition in quiet with speech in noise assessment in the routine audiologic test battery. Ear Hear. 2023;44(6):1548–1561. 10.1097/AUD.0000000000001409 PMC10583951 37703127

[11] Jason Qian Z, Vaisbuch Y, Gianakas SP, et al Evaluation of asymmetries in speech-in noise abilities in audiologic screening for vestibular schwannoma. Ear Hear. 2023;44(6):1540–1547. 10.1097/AUD.0000000000001397 PMC10583925 37707393

[12] Smith ML, Winn MB, Fitzgerald MB. A large-scale study of the relationship between degree and type of hearing loss and recognition of speech in quiet and noise. Ear Hear. 2024;45(4):915–928. 10.1097/AUD.0000000000001484 PMC11175802 38389129

[13] Fitzgerald MB, Ward KM, Gianakas SP, et al Speech-in-noise assessment in the routine audiologic test battery: Relationship to perceived auditory disability. Ear Hear. 2024;45(4):816–826. 10.1097/AUD.0000000000001472 PMC11175785 38414136

[14] Harkrider AW, Hedrick MS. Acute effect of nicotine on auditory gating in smokers and non-smokers. Hear Res. 2005;202(1-2):114–128. 10.1016/j.heares.2004.11.009 15811704

[15] Kunnath AJ, Gifford RH, Wallace MT. Cholinergic modulation of sensory perception and plasticity. Neurosci Biobehav Rev. 2023;152(105323):105323. 10.1016/j.neubiorev.2023.105323 PMC10424559 37467908

[16] Noftz WA, Beebe NL, Mellott JG, Schofield BR. Cholinergic projections from the pedunculopontine tegmental nucleus contact excitatory and inhibitory neurons in the inferior colliculus. Front Neural Circuits. 2020;14:43. 10.3389/fncir.2020.00043 PMC7378781 32765226

[17] Kanel P, van der Zee S, Sanchez-Catasus CA, et al Cerebral topography of vesicular cholinergic transporter changes in neurologically intact adults: A [18F]FEOBV PET study. Aging Brain. 2022;2(100039):100039. 10.1016/j.nbas.2022.100039 PMC9028526 35465252

[18] van der Zee S, Müller MLTM, Kanel P, van Laar T, Bohnen NI. Cholinergic denervation patterns across cognitive domains in Parkinson’s disease. Mov Disord. 2021;36(3):642–650. 10.1002/mds.28360 PMC7988481 33137238

[19] Nojszewska M, Pilczuk B, Zakrzewska-Pniewska B, Rowińska-Marcińska K. The auditory system involvement in Parkinson disease: Electrophysiological and neuropsychological correlations. J Clin Neurophysiol. 2009;26(6):430–437. 10.1097/WNP.0b013e3181c2bcc8 19952569

[20] Künzel T, Wagner H. Cholinergic top-down influences on the auditory brainstem. eNeuroforum. 2017;23(1):35–44.

[21] Lin FR, Pike JR, Albert MS, et al Hearing intervention versus health education control to reduce cognitive decline in older adults with hearing loss in the USA (ACHIEVE): A multicentre, randomised controlled trial. Lancet. 2023;402(10404):786–797. 10.1016/S0140-6736(23)01406-X PMC10529382 37478886

[22] Delano-Wood L, Bondi MW, Sacco J, et al Heterogeneity in mild cognitive impairment: Differences in neuropsychological profile and associated white matter lesion pathology. J Int Neuropsychol Soc. 2009;15(6):906–914. 10.1017/S1355617709990257 PMC3034688 19891820

[23] Metherate R, Weinberger NM. Cholinergic modulation of responses to single tones produces tone-specific receptive field alterations in cat auditory cortex. Synapse. 1990;6(2):133–145. 10.1002/syn.890060204 2237776

[24] Folmer RL, Vachhani JJ, Theodoroff SM, Ellinger R, Riggins A. Auditory processing abilities of Parkinson’s disease patients. Biomed Res Int. 2017;2017:2618587. 10.1155/2017/2618587 PMC5435898 28546963

[25] Lelo de Larrea-Mancera ES, Correa-Medina EI, Padilla-Bustos K, et al A characterization of central auditory processing in Parkinson’s disease. J Parkinsons Dis. 2024;14(5):999–1013. 10.3233/JPD-230458 PMC11307037 39031381

[26] Rajai Firouzabadi S, Mohammadi I, Golsorkh H, et al Hearing loss in Parkinson’s disease: A systematic review and meta-analysis. Mov Disord. 2026;41(5):1069–1080. 10.1002/mds.70206 41618507

[27] Zhang C, Burger RM. Cholinergic modulation in the vertebrate auditory pathway. Front Cell Neurosci. 2024;18:1414484. 10.3389/fncel.2024.1414484 PMC11220170 38962512

[28] Killion MC, Niquette PA, Gudmundsen GI, Revit LJ, Banerjee S. Development of a quick speech-in-noise test for measuring signal-to-noise ratio loss in normal-hearing and hearing-impaired listeners. J Acoust Soc Am. 2004;116(4 Pt 1):2395–2405. 10.1121/1.1784440 15532670

[29] Bohnen NI, Roytman S, van der Zee S, et al A multicenter longitudinal study of cholinergic subgroups in Parkinson disease. Nat Commun. 2025;16:5655. 10.1038/s41467-025-60815-0 PMC12219741 40593607

[30] Pichora-Fuller MK, Singh G. Effects of age on auditory and cognitive processing: Implications for hearing aid fitting and audiologic rehabilitation. Trends Amplif. 2006;10(1):29–59. 10.1177/108471380601000103 PMC4111543 16528429

[31] Bohnen NI, Albin RL. The cholinergic system and Parkinson disease. Behav Brain Res. 2011;221(2):564–573. 10.1016/j.bbr.2009.12.048 PMC2888997 20060022

[32] Müller MLTM, Bohnen NI. Cholinergic dysfunction in Parkinson’s disease. Curr Neurol Neurosci Rep. 2013;13(9):377. 10.1007/s11910-013-0377-9 PMC3991467 23943367

[33] Colangelo C, Shichkova P, Keller D, Markram H, Ramaswamy S. Cellular, synaptic and network effects of acetylcholine in the neocortex. Front Neural Circuits. 2019;13:24. 10.3389/fncir.2019.00024 PMC6473068 31031601

[34] Steinebach J, Reichenbach T. Attention to speech modulates distortion product otoacoustic emissions evoked by speech-derived stimuli in humans. Front Neurosci. 2026;20:1756386. 10.3389/fnins.2026.1756386 PMC12989502 41847227

[35] Wu JS, Yi E, Manca M, et al Sound exposure dynamically induces dopamine synthesis in cholinergic LOC efferents for feedback to auditory nerve fibers. Elife. 2020;9:e52419. 10.7554/eLife.52419 PMC7043886 31975688

[36] Moleti A, Minniti T, Viziano A, et al Enhanced suppression of otoacoustic emissions by contralateral stimulation in Parkinson’s disease. JASA Express Lett. 2023;3(10):104401. 10.1121/10.0021187 37787697

[37] Neilson LE, Reavis KM, Wiedrick J, Scott GD. Hearing loss, incident Parkinson disease, and treatment with hearing aids. JAMA Neurol. 2024;81(12):1295–1303. 10.1001/jamaneurol.2024.3568 PMC11581660 39432289

[38] Garasto E, Stefani A, Pierantozzi M, et al Association between hearing sensitivity and dopamine transporter availability in Parkinson’s disease. Brain Commun. 2023;5(2):fcad075. 10.1093/braincomms/fcad075 PMC10065189 37006327

[39] Wiggins IM, Stacey JE, Naylor G, Saunders GH. Relationships between subjective and objective measures of listening accuracy and effort in an online speech-in-noise study. Ear Hear. 2025;46(5):1197–1209. 10.1097/AUD.0000000000001662 PMC12352569 40117131

