# Supplementary material for: Cholinergic contributions to speech-in-noise processing in Parkinson’s disease
Source: Brain Commun. 2026 Oct 8;8(5):fcag384. doi: 10.1093/braincomms/fcag384 (PMC13696961; doi:10.1093/braincomms/fcag384)
Supplement: Supplementary file 1 [file fcag384_supplementary_data.pdf]

Supplementary Materials

Imaging Preprocessing Methods

FreeSurfer (v.7.4.1) was used to segment structural MRI images. The cerebral white matter mask obtained from the FreeSurfer segmentation was truncated below the highest point of the lateral ventricle and eroded with a 3mm radius sphere to obtain the reference region mask for processing the [18F]-FEOBV PET data. ANTsPyNet software library was used to perform brain extraction and ANTsPyX was used to generate a study-specific MRI template. ANTsPyX was used to compute a non-linear warp from each of the skull-stripped MRI images to the study-specific template.

The upper supratentorial white matter served as a reference region for quantification of vesicular acetylcholine transporter (VAChT) binding. For PD participants, VAChT PET imaging was conducted in the dopaminergic medication "on" state. Six dynamic imaging frames were co-registered to the first frame to reduce the influence of motion during the scan. The dynamic imaging frames were subsequently averaged and a rigid body transformation was computed from the PET image to the MRI image. The inverse rigid body transformation was applied to the reference region mask with nearest neighbor interpolation to bring it to PET space. The averaged dynamic image was subsequently divided by the mean value of the voxels belonging to the reference region in order to obtain an equilibrium distribution volume ratio (DVR) parametric

PET image (Chou et al. 2025). The inverse rigid body transform was used to warp the full set of FreeSurfer labels to PET space where region-based voxelwise partial volume correction was subsequently performed using the PETPVC software package (Thomas et al. 2016). The rigid-body transform to MRI space was composited with the non-linear warp to study-specific template space and applied to the parametric PET images to bring them into template space.

References

1. Chou, Kelvin L., Prabesh Kanel, Miriam van Emde Boas, Stiven Roytman, Giulia Carli, Roger L. Albin, and Nicolaas I. Bohnen. 2025. “Cholinergic System Changes in Dopa-Unresponsive Freezing of Gait in Parkinson’s Disease.” Movement Disorders: Official Journal of the Movement Disorder Society, April. https://doi.org/10.1002/mds.30196.
2. Thomas, Benjamin A., Vesna Cuplov, Alexandre Bousse, Adriana Mendes, Kris Thielemans, Brian F. Hutton, and Kjell Erlandsson. 2016. “PETPVC: A Toolbox for Performing Partial Volume Correction Techniques in Positron Emission Tomography.” Physics in Medicine and Biology 61 (22): 7975–93.
